# Investigation of Malocclusion and Associated Factors in Preschoolers: A Cross-Sectional Questionnaire Study

**DOI:** 10.3390/epidemiologia5020019

**Published:** 2024-06-06

**Authors:** Amanda Araújo de Carvalho, Tatiana Frederico de Almeida, Maria Beatriz Barreto de Sousa Cabral, Maria Cristina Teixeira Cangussu

**Affiliations:** Departamento de Odontologia Social e Pediátrica, Universidade Federal da Bahia (UFBA), Araújo Pinho Street, 6º Floor, 62 Canela, Salvador 41110-150, BA, Brazil; amanda.carvalho@ufba.br (A.A.d.C.); tfa@ufba.br (T.F.d.A.); mbscabral@ufba.br (M.B.B.d.S.C.)

**Keywords:** malocclusion, COVID-19, preschool, epidemiology

## Abstract

This study aims to describe the prevalence of malocclusion and identify associated factors in preschool children. Completed in 2022–2023, this cross-sectional study included 523 children aged 26 to 80 months in municipal schools in Salvador. An oral examination was carried out on the children, and a questionnaire was self-administered by the parents. Descriptive analyses and multivariate logistic regression (the backward method, *p*-value ≤ 0.05, 95% CI) were conducted. The majority of children were female (51.82%), over 54 months old (52.2%), Black or mixed race (90.63%), and not affected by COVID-19 (92.35%). The prevalence of malocclusion was 43.21%, with open bite as the most common condition. There was a significant association between malocclusion and screen time (OR: 1.34; *p*: 0.116; CI: 1.0–1.94), physical/psychological aggression (OR: 2.55; *p*: 0.031; CI: 1.0–5.98), consumption of ultra-processed foods (OR: 1.77; *p*: 0.003; CI: 1.22–2.57), digital suction (OR: 3.1; *p*: 0.001; CI: 1.56–6.16), and the habit of biting objects (OR: 1.56; *p*: 0.121; CI: 1.0–2.73). The promotion of comprehensive health in early childhood and psychosocial interventions are recommended, aiming to reduce screen time, aggression, consumption of ultra-processed foods, thumb sucking, and the habit of biting objects to prevent malocclusion.

## 1. Introduction

Malocclusion consists of a misalignment in the relationship between teeth and the growth and development of facial bones [1]. It is considered a public health problem in the preschool age group [2], with the potential to interfere with chewing, phonetics, and aesthetics. This condition has a multifactorial etiology, encompassing genetic and behavioral aspects [3], and is proven to be associated with factors such as non-nutritive sucking habits (pacifier and digital sucking), reduced breastfeeding duration, and socioeconomic aspects [4,5,6]. Its worldwide prevalence in primary dentition reaches an average of 54%, while in America, it reaches 53% [7]. In Brazil, specifically, according to the last national survey (SB BRASIL 2010), a prevalence of 65.5% was identified [8].

With the beginning of the second decade of the 2000s, significant changes have been observed, mainly because of the great flow of information, technological innovations, and the COVID-19 pandemic. In addition to the impact of the public health crisis and the physical impact, the pandemic had an intense impact on the dynamics of societies and affected the social, economic, and psychological spheres, especially for preschoolers aged 2 to 6 [9]. This entire context represented a significant risk to child development because of the chances of illness, social isolation, and increased instability in the family environment. Thus, there was a compromise in the cognitive, mental, and physical development of babies [10]. During this period, children’s oral health was heavily impacted, especially as a result of reduced access to health services because of lockdowns and delays in treatments. As a consequence, there were significant changes in the diagnosis of tooth decay, tooth retention, and malocclusion [11].

It is coherent to mention the relevant role of family experiences and stress mainly in the sphere of parental care [12]. As a consequence of sudden changes in family dynamics with the COVID-19 pandemic, changes in children’s eating, sleeping, and sociability routines have also been observed with greater intensity since 2020 [13,14]. Furthermore, a higher frequency of diagnoses of psychological distress in children was observed, with reports of exacerbated childhood anxiety, fear, and hyperactivity [15,16]. In addition, there has been a significant increase in reports of physical and psychological abuse and exposure to screen time [17,18]. Therefore, it is important to consider the family context in this recent global panorama as a modulator of experiences with great potential repercussions on children’s emotional regulation, habits, memory, and learning [19].

So, the hypothesis is supported by the development of deleterious habits and behaviors that contribute unusually to the occurrence of malocclusion, triggered by adverse social and family contexts, experiences of emotional stressors, and the need for emotional comfort. In addition to the above, it should be noted that the occurrence of the pandemic that began in March 2020 enabled intense financial instability and unemployment for many families, which may represent new challenges that can influence the prevalence of malocclusion. Therefore, there is a gap with a lack of studies in the current literature that cover the relationship between the occurrence of malocclusion in early childhood and current social contexts. It is also important to highlight the importance of understanding how recent social and behavioral changes can affect the oral and comprehensive health of infants. More than that, the study of these aspects makes early intervention possible and, consequently, contributes to a better quality of life for affected children. For that reason, a targeted study allows the development of targeted and appropriate interventions to promote comprehensive health in the early childhood period, with major gains in terms of the collective and individual health of children.

Thus, this study’s objective is to describe the prevalence of malocclusion and identify the emergence of associated factors in the current context in the preschool age group (26–80 months) in Salvador, Bahia.

## 2. Materials and Methods

This is a cross-sectional study that assessed the oral health status of preschool children in Salvador, Bahia, from 2022 to 2023. This research was approved by the Research Ethics Committee of the School of Dentistry at the Federal University of Bahia (UFBA)—CAAE: 60817222.6.0000.5024.

The study population consisted of children aged 26 to 80 months old, whether exposed to COVID-19 infection or not, who attended municipal public daycares. Inclusion criteria included children with a minimum age of 24 months and maximum age of 83 months old, providing consent to participate, and presenting an Informed Consent Form (ICF) signed by their parents or guardians. Exclusion criteria were age beyond or below the established age range, having a disease or condition that prevented examination, not providing consent, and lacking authorization from parents or guardians via an ICF signature.

Municipal daycares were chosen for this study based on convenience in the following districts of Salvador, Bahia: Barra, Brotas, Boca do Rio, Itapoã, Cabula, and Pau da Lima. Authorization was obtained from the Municipal Secretary of Education of Salvador, Bahia, to conduct this research in the mentioned daycares.

To determine the sample size with the minimum n, a calculation was carried out assuming an event proportion of 30% in the group and a minimum risk ratio of 1.3, according to Almeida et al. [20] and Carvalho et al. [21] in terms of the prevalence of the studied event. Furthermore, a power of 80% was established, and a significance level of 95% with a design error of 1.5. Thus, a minimum necessary sample of 388 preschoolers was identified.

Data collection instruments included a structured questionnaire for self-administration by parents/guardians and an oral examination form focusing on malocclusion. The questionnaire covered the following aspects: 1 = sociodemographic aspects such as age, gender, the mother’s education level (illiteracy up to post-graduation), household aspects (number of rooms, number of inhabitants in the home, and security of the place of residence), family income (in minimum wages), impact on family income during the pandemic (yes or no), adherence to social distancing (no adherence, parcial adherence, total adherence), and the child’s history of COVID-19 infection (yes or no); 2 = dietary habits and food security such as consumption of fruits, vegetables, processed foods (everyday, sometimes, or never), and changes in diet during the pandemic (less or more access, type of most consumed food); 3 = the child’s daily habits and lifestyle such as sleep quality (poor or healthy), screen time (less than 2 h or more than 2 h), outdoor activities during the pandemic (yes or no), feelings of anxiety or fear and experiences with physical or psychological violence during the pandemic (yes or no); 4 = the child’s oral habits, and access to health services such as brushing frequency (at least once a day—yes or no), presence of bruxism/clenching during the pandemic (yes or no), digital sucking (yes or no), pacifier sucking (yes or no), and nail-biting(yes or no) and object-biting habits (yes or no) and the need for dental care during the pandemic (yes or no, type of dental care, and the reason for seeking it).

Data collection, including questionnaire delivery and oral examination, took place on the daycare premises and was conducted by trained and calibrated dental surgeons and professors from the School of Dentistry at UFBA, who have 5 to 25 years of experience in surveys. Inter-examiner and intra-examiner agreements were verified through the kappa test (above 0.9). Examinations were conducted according to the biosafety standards of the World Health Organization (WHO), using Personal Protective Equipment (disposable gown, cap, N-95 respirator, protective eyewear, face shield, gloves) and wooden spatulas. For optimal assessment, children were examined while sitting on chairs under natural light. Malocclusion was assessed through an oral examination based on canine classification (classes I, II, and III) and the WHO malocclusion index, overjet, overbite, anterior open bite, and anterior or posterior crossbite solely through visual assessment at the crown level. The oral examination was only observational, and wooden spatulas were used because of the need to remove mucous membranes for better visualization. No intervention was carried out.

Data from questionnaires and oral examination forms were tabulated in an Excel database, and statistical analysis was performed using the software Minitab, version 14 (Solutyons Analytics; State College, PA, USA). Descriptive analyses of categorical and continuous variables and an exploratory analysis were conducted to identify potential factors associated with dental malocclusion.

Chi-square tests were performed with a significance level of 5% to identify dependent and independent variables associated with malocclusion. Subsequently, a bivariate and multivariate exploratory analysis was conducted to establish risk or protective variables and the risk ratio for malocclusion in children aged 2 to 6 years.

Multivariate logistic regression analysis was performed using the backward method, with a 95% confidence interval, based on selected variables from the bivariate model with a significance level of up to 20%. Variables exhibiting collinearity were excluded to enhance the predictive value of multivariate analysis.

## 3. Results

This study included 523 children aged 26–80 months (mean age of 54 months), residing in the city of Salvador, Bahia, and enrolled in municipal public daycares. The sociodemographic characterization of the study population is described in Table 1. Overall, 51.82% of the children were female, with the majority of the study population being older than 54 months (52.2%) and predominantly Black or mixed race (90.63%). Regarding the mothers, 51.82% were aged up to 31 years, and the majority had completed high school education (80.31%).

Regarding housing conditions, it was observed that 65.77% of the children lived with up to three people in the same household, with most of them having a maximum of five rooms (77.25%). Only 18.36% of the families considered their place of residence dangerous. Regarding income, 74% of the families lived on up to one minimum wage (MW), the majority (69.22%) reported financial impacts due to the COVID-19 pandemic, and 66.35% received emergency assistance during this period (Table 1).

Similarly, Table 1 describes the dietary habits and main impacts of the pandemic on preschoolers. Specifically for dietary habits, it was observed that 96.65% of children had meals at the daycares they attended, with the majority consuming fruits or vegetables weekly (93.12%) and snacks or processed foods (99.24%). Most parents (75.14%) did not report significant impacts on their children’s diet due to the pandemic; however, 58.89% of the population had reduced access to food during this period. Approximately 48% of parents reported an increase in the consumption of healthy foods during the pandemic, and 17.97% reported increased consumption of ultra-processed foods during this period (Table 1).

Additionally, 20.08% of children in this study had reduced sleep quality during the pandemic, along with reports of increased screen time by 60.61%. Overall, 76.29% of the evaluated children spent more than 2 h using screens during this period, and 62.72% did not have access to remote classes during the suspension of in-person activities in daycares and schools. Outdoor activities did not occur for 62.91% of the children during the pandemic, and experiences of fear and/or anxiety were reported in 22.75% of the children. Reports of verbal or physical aggression by adults towards children occurred in approximately 5% of the study population (Table 1).

Table 2 contains a description of the oral habits and conditions of the study population. Overall, 76.29% of the preschoolers brush their teeth with parents or guardians, and 69.6% use fluoridated toothpaste. During the pandemic, deleterious habits such as bruxism or clenching, digital-sucking, pacifier-sucking, nail-biting, and object-biting habits occurred in the children with a prevalence of 19.69%, 8.03%, 6.88%, 20.46%, and 12.24%, respectively. Approximately 45% of the children continued these habits after the pandemic (Table 2).

**Figure 1:**
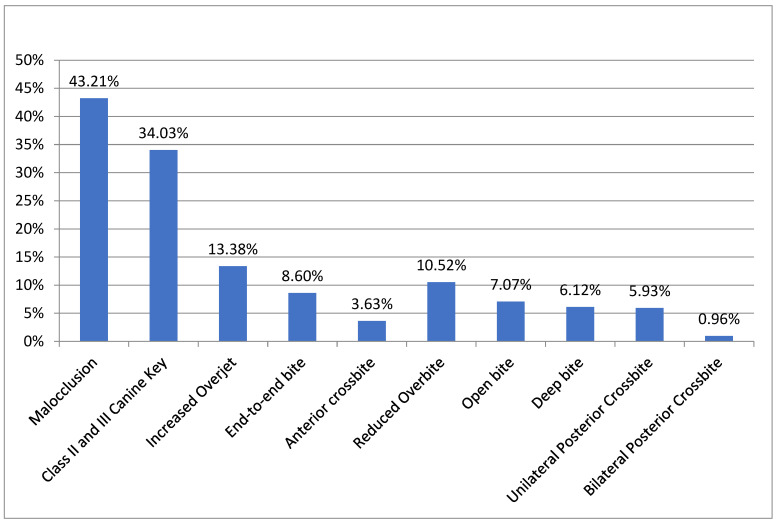
Prevalence of malocclusion in preschoolers in Salvador-Ba, 2022–2023—N = 523.

The prevalence of malocclusion in the preschoolers is presented in Figure 1, as well as the occurrence of specific occlusion alterations in this group. A prevalence of 43.21% of malocclusion was observed in the study population. The canine classification as Classes II and III occurred in 34.03% of the preschoolers. Overjet was normal in 74.38% and increased in 13.38% of the study population. There was a prevalence of 8.6% for end-to-end occlusion and 3.63% for anterior crossbite. When evaluating overbite, it was normal in 76.29% of the population, reduced in 10.52%, open in 7.07%, and deep in 6.12%. Posterior crossbite was absent in 93.12%, unilateral in 5.93%, and bilateral in approximately 1% of the children (Figure 1).

For the bivariate analysis, the distribution of variables of interest was conducted according to the presence and absence of malocclusion. The variables were categorized based on sociodemographic aspects, dietary habits and pandemic impacts, and oral habits, corresponding to Table 3. Adopting a maximum *p*-value of 0.20, the following variables were selected to compose the final multivariate model: sociodemographic including “mother’s age” and “level of social distancing”; dietary habits and pandemic impacts including “higher consumption of healthy foods”, “increased screen time during the pandemic”, and “verbal or physical aggression by adults”; and oral habits including “digital-sucking” and “object-biting”.

As shown in Table 4, through multivariate logistic regression analysis, a statistically significant association is observed between the presence of malocclusion and the consumption of ultra-processed foods, where higher consumption of these foods represented a 1.77 times higher chance of developing malocclusion compared with children with healthy dietary habits. Regarding increased screen time, this habit represented a 1.34 times greater chance of developing malocclusion compared with children who used screens for a shorter time. Concerning the family environment, the experience of physical or psychological aggression inflicted by an adult at home was shown to be a potential factor associated with malocclusion in preschoolers at 2.55 times. For oral habits, digital sucking presented an Odds Ratio = 3.1 for the occurrence of malocclusion, while the habit of biting objects appeared as a potential risk factor at 1.56 times. The maintenance of variables “increased screen time” and “biting objects” occurred because of the theoretical model despite the presented significance threshold.

## 4. Discussion

Malocclusion was the oral condition adopted as the main focus of the present study. According to the findings, the prevalence of this condition was 43.21% among the 523 preschoolers examined. Among the types of malocclusion assessed, open bite showed the highest prevalence, affecting 7% of the study population. Furthermore, a statistically significant association was demonstrated between the occurrence of malocclusion and deleterious habits such as digital-sucking and object-biting, consumption of ultra-processed foods, increased screen time during the pandemic period, and experience of physical or psychological aggression.

It is relevant to note that the prevalence of malocclusion is heterogeneous across the country, showing differences depending on the study population, sample size, chosen region, and adopted assessment instruments. The last Brazilian national oral health survey with published data, SB Brazil, was conducted in 2010 and identified a prevalence of malocclusion in 65.5% of 5-year-old children [8]. However, there is considerable variation in the country, with figures ranging from 53.4% [22], 57.3% [23], to 63.3% [24] in the literature. In a meta-analysis conducted in 2020, however, the global average identified was 54% [7].

Specifically for the city of Salvador-Ba, a growing trend in the prevalence of malocclusion is observed, as signaled by the comparison between studies conducted in the locality. Almeida et al. (2005) identified a prevalence of 37.6% [20], Carvalho et al. (2019) found 40.46% [21], and the present study identified 43.21%. It is believed that this increasing trend in the occurrence of malocclusion in preschoolers over time signals a lack of effectiveness in preventive, promotional, and educational health measures for preschoolers and their families.

Since March 2020, mainly because of the COVID-19 pandemic, preschoolers have been particularly affected by the panorama of social isolation, the impossibility of activities outside the home, and the interruption of face-to-face activities in schools [25]. In the present study, increased screen time during the pandemic period was linked to the occurrence of malocclusion, with a 1.34 times higher chance compared with screen time up to the 60 min recommended by the WHO [26]. This increase in time spent using screens occurred mainly as a form of entertainment for children and as a remediation of negative emotions such as anxiety and distress [27]. In line with this finding, Silvério et al. (2023) identified an association between adverse emotional experiences, imbalance in the family environment, and excessive screen use [25].

Thus, preschoolers are observed to be vulnerable to developing habits that provide ways to overcome the increasing emotional demands of this period. This point is reinforced by Silvério et al. (2023), who reported a higher occurrence of regressive behaviors during the pandemic period from 2020 to 2022. Therefore, excessive screen use may have been accompanied by an increase in the frequency, intensity, and duration of deleterious habits that facilitate the occurrence of malocclusion [25].

Regarding non-nutritive sucking habits, there is evidence in the literature of their association with a higher prevalence of malocclusion in the preschool age group, especially because of their capacity for emotional compensation in the face of emotional deficiencies and early weaning [21]. In the present study, digital sucking was associated with the occurrence of malocclusion in the age group of 2 to 6 years, with a statistically significant relationship with a 3.1 times higher chance. This result can be justified by the experiences of feelings such as anxiety and sadness in children during this period [25] and the consequent need to meet this demand.

On the other hand, the present study did not identify an association between the occurrence of malocclusion and the habit of pacifier sucking. This result contradicts the extensive scientific evidence of a statistically significant association between the development of malocclusion and pacifier sucking in preschoolers, as observed in cross-sectional studies conducted by Carvalho et al. (2021), Pegoraro et al. (2022), and Davidopoulou et al. (2022) [21,22,28]. Therefore, there is a possibility that the finding was underestimated in the present study because of the interference of the frequency, intensity, and duration triad of pacifier sucking by the children who composed the study population, where a prevalence of the habit was identified in 6.88%.

Furthermore, the habit of object biting was also recognized with statistical significance in its association with the occurrence of malocclusion in the present study. A 1.56 times higher possibility of malocclusion presence was observed for children exhibiting the deleterious habit. This finding is consistent with the results found by Xu et al. (2022) in a cross-sectional study, which identified a relationship between object biting and malocclusion at age 6 [29].

From another perspective, temporomandibular disorders such as bruxism affect the temporomandibular joint, muscles involved in chewing, and adjacent structures [30]. In this sense, both the habit of object biting and experiences of anxiety are associated with sleep bruxism in the 7–15 years age group and preschoolers [31]. Conversely, the presence of parafunction enables the occurrence of malocclusion in the studied age group, as observed by Carvalho et al. (2021) [21] and by Ghafournia and Tehrani in 2012 [32]. However, in a systematic review conducted by Thijs et al. (2021), a clear relationship between malocclusion and the habit of object biting and bruxism was not identified [33]. These findings highlight that the relationship among the habits of object biting, bruxism, and malocclusion has not reached a consensus in the literature and signals the need for further investigation in this regard.

Furthermore, the consumption of ultra-processed foods by the preschoolers presented statistical significance with malocclusion in this group, with a 1.77 times higher chance of occurrence of the condition. This point relates to the aspect of food security, which in 2023 was present in only 72.4% of Brazilian households, according to the Brazilian Institute of Geography and Statistics (IBGE). For the Northeast, this number was even lower, reaching only 61.2% of households [34]. Given this circumstance, it is understood that the ingestion of ultra-processed foods is favored, especially for households with food insecurity and socioeconomic difficulties, and changes in the dynamics of populations that occurred between 2020 and 2022, mainly because of the pandemic, should be rescinded.

Moreover, the consistency of ingested foods is an important factor in the development of the stomatognathic system and normal occlusion [35]. This theory is corroborated by Kuninori et al. (2014) and Consolação Soares (2016), who observed that a softer food consistency enables lower chewing efficiency [36,37]. It should also be emphasized that Yeung et al. (2024) recognized the interrelation between the existence of non-nutritive sucking habits and a preference for softer consistent foods [38]. In this sense, Corrêa et al. (2018) observed that reduced chewing efficiency was more common in children with malocclusion [39]. This fact is exacerbated according to the severity of malocclusion [40]. However, conversely, Lima et al. (2018) did not identify a statistically significant relationship between the occurrence of malocclusion and dietary patterns [41].

From another perspective, the experience of physical or psychological aggression was also significantly associated with malocclusion in preschoolers, with a 2.55 times higher chance of the condition occurring. This finding may be related to the relevant role of emotional regulation exerted by the family on children, and, in this sense, a family environment where violence occurs enables emotional instabilities in this group [42]. However, once socioeconomic vulnerabilities are established in the home, there is a greater predisposition and vulnerability to the occurrence of domestic violence [43]. It is believed that the context itself potentially triggers emotional responses in children that may favor the adoption of deleterious habits such as non-nutritive sucking, compensatory food consumption with comfort foods like ultra-processed foods, and parafunctions such as bruxism.

The potential limitations of the present study should be considered, such as biases related to confounding during the completion of self-administered questionnaires. In addition, the study population was a convenience sample. Although the data collection sites belonged to different health districts of the city with different socioeconomic indicators, there was conditioning to the receptivity and availability of invited municipal daycare centers. Because of the relevance of the topic and various health determinants, especially for the preschool population, it is suggested that new studies should be conducted on representative study populations. When considering recent changes in population dynamics, especially after the COVID-19 pandemic, the potential behavioral, emotional, and physical changes occurring in the preschool population should be highlighted. The present study is one of the first to evaluate which new potential factors associated with malocclusion in this age group may have emerged or remained in response to the context in which infants are currently inserted. Furthermore, our findings are relevant to clinical practice and the development of comprehensive and appropriate public health policies related to the occurrence of malocclusion in preschoolers.

## 5. Conclusions

The findings of the present study indicate an increasing trend in the prevalence of malocclusion in preschool age compared with previous studies carried out in the same location. Such results have great relevance for children’s public health. Although some factors potentially associated with the occurrence of malocclusion demonstrate persistence, it is important to highlight that the new possible associations identified—such as screen time, consumption of ultra-processed foods, and experience of physical or psychological violence—require the adaptation of child public health efforts. Actions that promote comprehensive health in early childhood are recommended, with an emphasis on psychosocial interventions, to reduce and prevent any changes that may facilitate the occurrence of malocclusion. Furthermore, additional studies are needed to enable the understanding and identification of new factors associated with malocclusion.

## Figures and Tables

**Table 1:** Sociodemographic characterization, eating habits, and impacts of the pandemic on preschoolers in Salvador-Ba, 2022–2023—N = 523.

	N	%
Skin color		
Black	474	90.63
Others	49	9.37
Age (months)		
Under to 54 months	250	47.80
Over 54 months	273	52.20
Gender		
Female	271	51.82
Male	252	48.18
Child’s history of COVID-19		
Yes	40	7.65
No	483	92.35
Mother’s age		
Up to 31 years	271	51.82
Over 31 years	252	48.18
Mother’s education		
Up to secondary education	420	80.31
Higher than secondary education	103	19.69
Number of people in the household		
Up to three people	344	65.77
Over three people	179	34.23
Number of rooms in the home		
Up to five rooms	404	77.25
Over five rooms	119	22.75
Dangerous place of residence		
Yes	96	18.36
No	427	81.64
Family income		
Up to one MW *	387	74
Over one MW *	136	26
Level of social distancing		
Total or almost total	352	67.0
Only for work or no changes	171	32.70
Impact of the pandemic on family income		
No	161	30.78
Yes	362	69.22
Receiving emergency aid		
Yes	347	66.35
No	176	33.65
Meal at daycare		
Yes	495	96.65
No	28	5.35
Weekly fruit or vegetable intake		
Yes	487	93.12
No	36	6.88
Weekly intake of processed foods		
Yes	519	99.24
No	4	0.76
Impact of the pandemic on nutrition		
Yes	130	24.86
No	393	75.14
Type of impact of the pandemic on nutrition		
Reduced access	308	58.89
Increased access	215	41.11
Increased access to:		
Nothing	178	34.03
Processed foods	94	17.97
Healthy foods	251	47.99
Reduced sleep quality		
Yes	105	20.08
No	418	79.92
Increased screen time		
Yes	317	60.61
No	206	39.39
Screen Time		
Up to 2 h	124	23.71
Over 2 h	399	76.29
Access to remote classes		
Yes	195	37.28
No	328	62.72
Outdoor activities		
Yes	194	37.09
No	329	62.91
The feeling of anxiety or fear		
Yes	119	22.75
No	404	77.25
Persistent feelings of anxiety or fear		
Yes	42	8.03
No	481	91.97
Child’s experience of physical or verbal aggression		
Yes	26	4.97
No	497	95.03

* MG = minimun wage; self-elaboration.

**Table 2:** Oral habits and conditions in preschool children in Salvador-Ba, 2022–2023—N = 523.

	N	%
Teeth brushing		
Accompanied	399	76.29
Alone	124	23.71
Use of fluoride toothpaste		
Yes	364	69.6
No	159	30.4
Bruxism or tightening		
Yes	103	19.69
No	420	80.31
Digital suction		
Yes	42	8.03
No	481	91.97
Pacifier suction		
Yes	36	6.88
No	487	93.12
Biting nails		
Yes	107	20.46
No	416	79.54
Biting objects		
Yes	64	12.24
No	459	87.76
Persistence of habits		
Yes	235	44.93
No	288	55.07

Self-elaboration.

**Table 3:** Distribution of malocclusion according to sociodemographic covariates, eating habits, impacts of the pandemic, and oral habits in preschoolers in Salvador-Ba, 2022–2023—N = 523.

Malocclusion					
	Presence		Absent		*p*-Value
	N	%	N	%	
Gender					0.387
Female	122	53.98	149	50.17	
Male	104	46.02	148	49.83	
Age (months)					0.720
Up to 54 months	106	46.90	144	48.48	
Over 54 months	120	53.10	153	51.52	
Skin color					0.580
Black	203	89.82	271	91.25	
Other	23	10.18	26	8.75	
Child’s history of COVID-19					0.448
No	211	93.36	272	91.58	
Yes	15	6.64	25	8.42	
Mother’s age					**0.152**
Up to 31 years	109	48.23	162	54.55	
Over 31 years	117	51.77	135	45.45	
Mother’s education					0.580
Up to secondary education	179	79.20	241	81.14	
Higher than secondary education	47	20.80	56	18.86	
Number of people in the household					0.533
Up to three people	152	67.26	192	64.65	
Over three people	74	32.74	105	35.35	
Number of rooms in the home					0.240
Up to five rooms	169	74.78	235	79.12	
Over five rooms	57	25.22	62	20.88	
Dangerous place of residence					0.427
Yes	38	16.81	58	19.53	
No	188	83.19	239	80.47	
Family income					0.963
Up to one MW *	167	73.89	220	74.07	
Over one MW *	59	26.11	77	25.93	
Level of social distancing					**0.195**
Total or almost total	159	70.35	193	64.98	
Only for work or no changes	67	29.65	104	35.02	
Impact of the pandemic on family income					0.785
No	71	31.42	90	30.30	
Yes	155	68.58	207	69.70	
Receiving emergency aid					0.568
Yes	153	67.70	194	65.32	
No	73	32.30	103	34.68	
Meal at daycare					0.666
Yes	215	95.13	280	94.28	
No	11	4.87	17	5.72	
Weekly fruit or vegetable intake					0.394
Yes	208	92.04	279	93.94	
No	18	7.96	18	6.06	
Weekly intake of processed foods					0.783
Yes	224	99.12	295	99.33	
No	2	0.88	2	0.67	
Increased access to:					**0.032**
Nothing	91	40.27	87	29.29	
Processed foods	37	16.37	57	19.19	
Healthy foods	98	43.36	153	51.52	
Reduced sleep quality					0.424
Yes	49	21.68	56	18.86	
No	177	78.32	241	81.14	
Increased Screen time					**0.148**
Yes	145	64.16	172	57.9	
No	81	35.54	125	42.09	
Access to remote classes					0.551
Yes	81	35.84	114	38.38	
No	145	64.16	183	61.62	
Outdoor activities					0.212
Yes	77	34.07	117	39.39	
No	149	65.93	180	60.61	
The feeling of anxiety or fear					0.335
Yes	56	24.78	63	21.21	
No	170	75.22	234	78.79	
Persistent feelings of anxiety or fear					0.709
Yes	17	7.52	25	8.42	
No	209	92.48	272	91.58	
Child’s experience of physical or verbal aggression					**0.019**
Yes	17	7.52	9	3.03	
No	209	92.48	288	96.97	
Teeth brushing					0.742
Accompanied	174	76.99	225	75.76	
Alone	52	23.01	72	24.24	
Use of fluoride toothpaste					0.955
Yes	157	69.47	207	69.70	
No	69	30.53	90	30.30	
Bruxism or tightening					0.580
Yes	47	20.80	56	18.86	
No	179	79.20	241	81.14	
Digital suction					**0.001**
Yes	28	12.39	14	4.71	
No	198	87.61	283	95.29	
Pacifier suction					0.373
Yes	13	5.75	23	7.74	
No	213	94.25	274	92.26	
Biting Nails					0.252
Yes	41	18.14	66	22.22	
No	185	81.86	231	77.78	
Biting objects					**0.128**
Yes	22	9.73	42	14.14	
No	204	90.27	255	85.86	
Persistence of habits					0.540
Yes	105	46.46	130	43.77	
No	121	53.54	167	56.23	

* MG = minimum wage, self-elaboration. bold shows the variables used in multivariate model.

**Table 4:** Final model of the multivariate logistic regression analysis for malocclusion in preschool children in Salvador-Ba, 2022–2023—N = 523.

Variables	No Malocclusion OR *	With Malocclusion OR *	95% CI *	*p*-Value
Increased screen time	1.0	1.34	1.0–1.94	0.116
Child’s experience of physical or verbal aggression	1.0	2.55	1.0–5.98	0.031
Intake of processed foods	1.0	1.77	1.22–2.57	0.003
Digital suction	1.0	3.10	1.56–6.16	0.001
Biting objects	1.0	1.56	1.0–2.73	0.121

OR * = Odds Ratio; CI * = confidence interval; self-elaboration.

## Data Availability

The data presented in this article are available upon request from the authors.
